# High-Dose Astaxanthin Supplementation Suppresses Antioxidant Enzyme Activity during Moderate-Intensity Swimming Training in Mice

**DOI:** 10.3390/nu11061244

**Published:** 2019-05-31

**Authors:** Yingsong Zhou, Julien S Baker, Xiaoping Chen, Yajun Wang, Haimin Chen, Gareth W Davison, Xiaojun Yan

**Affiliations:** 1 School of Sports Sciences and Physical Education, Ningbo University, Ningbo 315211, China; zhouyingsong@nbu.edu.cn (Y.Z.); chenxiaoping@nbu.edu.cn (X.C.); 2 Institute for Clinical Exercise & Health Science, University of the West of Scotland, Lanarkshire, Scotland G72 OLH, UK; jsbaker@uws.ac.uk; 3 School of Marine Sciences, Ningbo University, Ningbo 315211, China; wangyajun@nbu.edu.cn (Y.W.); chenhaimin@nbu.edu.cn (H.C.); 4 Sport and Exercise Sciences Research Institute, Ulster University, Newtownabbey BT37 0QB, UK; gw.davison@ulster.ac.uk

**Keywords:** astaxanthin, antioxidant, oxidative stress, chronic exercise, physical adaption

## Abstract

Exercise-induced reactive oxygen and nitrogen species are increasingly considered as beneficial health promotion. Astaxanthin (ASX) has been recognized as a potent antioxidant suitable for human ingestion. We investigated whether ASX administration suppressed antioxidant enzyme activity in moderate-intensity exercise. Seven-week-old male C57BL/6 mice (*n* = 8/group) were treated with ASX (5, 15, and 30 mg/kg BW) combined with 45 min/day moderate-intensity swimming training for four weeks. Results showed that the mice administrated with 15 and 30 mg/kg of ASX decreased glutathione peroxidase, catalase, malondialdehyde, and creatine kinase levels in plasma or muscle, compared with the swimming control group. Beyond that, these two (15 and 30 mg/kg BW) dosages of ASX downregulated gastrocnemius muscle erythroid 2p45 (NF-E2)-related factor 2 (Nrf2). Meanwhile, mRNA of Nrf2 and Nrf2-dependent enzymes in mice heart were also downregulated in the ASX-treated groups. However, the mice treated with 15 or 30 mg/kg ASX had increased constitutive nitric oxidase synthase and superoxide dismutase activity, compared with the swimming and sedentary control groups. Our findings indicate that high-dose administration of astaxanthin can blunt antioxidant enzyme activity and downregulate transcription of Nrf2 and Nrf2-dependent enzymes along with attenuating plasma and muscle MDA.

## 1. Introduction

For decades, regular, nonexhaustive physical exercise has been considered beneficial for improving health and physical fitness. Many studies show that chronic physical exercise can prevent several chronic diseases (e.g., cardiovascular disease, diabetes, cancer, hypertension, obesity, depression, and osteoporosis) and premature death. However, during high-intensity exercise, reactive oxygen and nitrogen species (RONS) are yielded simultaneously, which may damage important macromolecules such as lipids, protein, and DNA [1,2]. That said, organisms have evolved complicated endogenous antioxidant defense mechanisms to minimize the potential damage caused by increased oxidative stress [3]. Key antioxidant enzymes, such as glutathione peroxidase (GPx), superoxide dismutase (SOD), and catalase (CAT), synergistically eliminate damaging free radical species. Two nuclear factors, erythroid 2p45 (NF-E2)-related factor 2 (Nrf2) and kelch-like ECH-associated protein 1 (Keap1), regulate the transcription of phase II detoxifying enzymes in accordance with oxidative stress, thereby maintaining cellular homeostasis in vivo [4,5]. Many Nrf2-dependent antioxidant enzymes, which have antioxidant-response elements (AREs) in untranslated gene regions, are activated by Nrf2. These enzymes embrace glutamate-cysteine ligase catalytic subunit (GCLC) and glutamate-cysteine ligase modifier subunit (GCLM), which are the first-rate limiting enzymes of glutathione synthesis [6,7]; NAD(P)H quinone dehydrogenase 1 (NQO-1), which prevents the one-electron reduction of quinones [8]; Heme oxygenase 1 (HMOX-1), which cleaves heme to form biliverdin [9]. Although an overabundance of RONS, generated within cells, can be counterproductive and overwhelm the endogenous antioxidant defense system, an optimal amount of RONS produced during exercise can be beneficial for exercise adaption and overall health [10,11,12]. Over the years, many studies have shown that oral administration of vitamin C or vitamin C and E complex decreases muscle mitochondrial biogenesis and hampers physical adaptations in endurance performance [13,14,15,16,17]. However, other studies have shown that antioxidant treatment does not eliminate the beneficial effects of exercise [18,19,20,21,22]. Therefore, the benefits of antioxidant supplementation in exercise training have become a controversial topic.

Astaxanthin (ASX) is a xanthophyll carotenoid, which is a fat-soluble red pigment found in several species, such as microalgae, crustacea, fish, and birds [23]. According to the previous studies, ASX can directly scavenge peroxyl (ROO•), alkoxyl (RO•), and singlet oxygen in a dose-dependent way, but does not directly affect transcription of antioxidant enzymes [24]. Many other studies have documented that dietary consumption of ASX can prevent or reduce the risk of various medical conditions in humans and animals [25]. In addition, long-term supplementation of ASX in mice also delays time to exhaustion during exercise [26,27,28,29,30]. Hence, ASX is considered a potent antioxidant utilized as a nutritional supplement for physical exercise participants [31]. Nevertheless, previous studies have revealed that antioxidant supplementation can blunt the expression of antioxidant enzymes; this may decrease muscle adaption capability following exercise [16,17]. As such, it is our contention and hypothesis that ingestion of high doses of the antioxidant ASX may blunt antioxidant enzymes in vivo during bouts of regular physical exercise. Therefore, the purpose of this study was to investigate the relationship between antioxidant status and the dosage of ASX supplementation using a moderate-intensity exercise mouse swimming model. 

## 2. Materials and Methods

### 2.1. Astaxanthin Source, Animals, and Experimental Design 

Astaxanthin samples (Cat. No. SML 0982) were purchased from Sigma-Aldrich Co. LLC (Saint Louis, MO, USA) and dissolved in olive oil, which was prepared in advance and stored at −20 °C. Forty male C57BL/6 mice (7 weeks old) with weights ranging from 20~25 g were obtained from the Experimental Animal Centre of Zhejiang Province, China, and four mice in each cage were housed in the Animal Centre of Ningbo University College of Medicine (Ningbo, China). All mice were acclimatized for one week in an air-conditioned (22 ± 2 °C and approximately 60% RH) room under a 12 h light/dark cycle (lights on from 07:30 to 19:30 h) with food and water provided *ad libitum*. The composition of mouse feed is listed in a Supplementary Materials.

The mice were randomly divided into five groups, consisting of a sedentary control (SEC) group, a swimming control (SWC) group, and three swimming plus ASX (SA) groups; each group was assigned eight mice. For eliminating the background effect of olive oil acting on mice, the mice in the SEC and SWC groups were orally administered 0.1 mL of olive oil by gavage each day. The mice in the three SA groups were orally treated with 0.1 mL of the mixture of ASX and olive oil by gavage and participated in a supplementation regime with dosages of ASX 5 mg/kg Body Weight (BW; SA5), 15 mg/kg BW (SA15), and 30 mg/kg BW (SA30), respectively. The supplemented quantity of ASX was based on published work [26,28]. However, some modifications were made for evaluating ASX concentrations in larger quantities during this experiment. All the mice were fed with an ASX mixture or olive oil two hours prior to the initiation of swimming training. 

**Figure 1:**
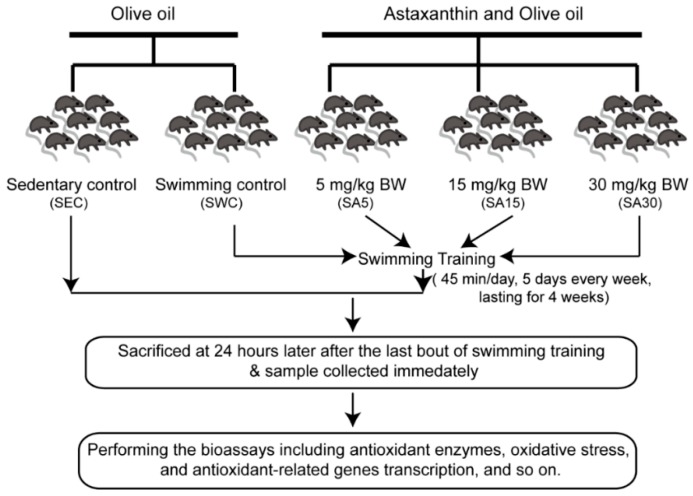
The experimental design and flow diagram for evaluation of astaxanthin.

The mice swimming model was based on previous research with slight modifications [32,33]. The mice in the swimming groups performed chronic swimming training in a heated water tank (31 ± 2 °C), five times a week for four weeks. No weight loads were used. During the four-week training period, the durations of the swimming tasks were gradually increased from an initial 10 min/time to a final level of 45 min/time, with an increasing duration of 5 min. After four weeks of training, all experimental mice were sacrificed at 24 h postexercise (Figure 1). The experimental protocol used was in accordance with the National Institutes of Health Guidelines for the Care and Use of Laboratory Animals and approved by the Ethical Committee of Animal Use and Protection at Ningbo University Health Science Centre. The handling of animals was also in accordance with the consensus author guidelines on animal ethics.

### 2.2. Sample Collection and Preparation

Blood samples were immediately collected from an eye socket vein and preserved in EDTA-tubes. The blood samples were centrifuged at 3000× *g* for 5 min, and the plasma aliquots were subsequently stored at −80 °C for biochemical analysis (enzyme activity and lipid peroxidation). The gastrocnemius muscles of the mice were isolated immediately, and each one was divided into two portions. One portion, used for total RNA extraction, was immersed in RNAlater reagent (Cat. No. AM7020, Ambion, city, country) and stored at −20 °C after one-night equilibration at 4 °C. The other portion was wrapped in aluminum foil and stored at −80 °C. The gastrocnemius muscle tissue used for the determination of enzyme activity or lipid peroxidation was homogenized in saline in an ice bath. The homogenated samples were centrifuged at 3500× *g* for 10 min and the supernatants were placed into new EP tubes and immediately stored at −80 °C for future analysis.

### 2.3. Antioxidant Enzymes and Malondialdehyde Assays 

Total superoxide dismutase (SOD) activity in plasma was determined using a superoxide dismutase (SOD) assay kit (Cat. No. A001-3, Nanjing Jiancheng Biotech Company, Nanjing, China). The glutathione peroxidase (GPx) activity both in plasma and muscle was assayed using a glutathione peroxidase assay kit (Cat. No. A005, Nanjing Jiancheng Biotech Company). Catalase (CAT) activity in muscle was measured using a catalase assay kit (Cat. No. A007-1, Nanjing Jiancheng Biotech Company). Malondialdehyde (MDA) concentrations in plasma or muscle tissue were obtained using a Microscale malondialdehyde assay kit (Cat. No. A003-2, Nanjing Jiancheng Biotech Company). All assays were performed and completed following the manufacturer’s instructions.

### 2.4. Nitric Oxide Synthase and Creatine Kinase Assays

Plasma nitric oxide synthase activity was assayed using the Nitric Oxide Synthase typed assay kit (Cat. No. A014, Nanjing Jiancheng Biotech Company). The kit was used to quantify total nitric oxide synthase (tNOS), inducible nitric oxide synthase (iNOS), and constitutive nitric oxide synthase (cNOS) activity. cNOS activity was calculated by the value of tNOS subtracted by that of iNOS. Plasma creatine kinase (CK) activity was measured by a creatine kinase assay kit (Cat. No. A032, Nanjing Jiancheng Biotech Company). All assays were conducted in accordance with the manufacturer’s instructions. 

### 2.5. Analysis of Nrf2 and Nrf2-Dependent Gene Transcription in Gastrocnemius and Heart

Total RNA was extracted from the gastrocnemius muscle or heart from different groups using TransZol Up Plus RNA Kit (*Cat.No. ER501*, Tansgen Biotech, Beijing, China), following the manufacturer’s instructions. Two micrograms of each total RNA sample aliquot was treated with RNase free DNase I (Takara, Dalian, China) and desalted before the first strand cDNA synthesis using RNeasy MinElute Cleanup Kit (Cat.No. 74204, QIAGEN, Germany). The first-strand cDNA was synthesized using HiFiScript gDNA Removal cDNA Synthesis kit (Cat.No. CW2582M, CWBIO, China). The quantitative PCR was performed using TB Green *Premix Ex Taq* II kit (Takara, Japan). To evaluate PCR efficiency, ten-fold serial dilutions of target gene plasmid cDNA, were used to create standard curves for each gene. The specific primers (5′ to 3′) were GCTCCTATGCGTGAATCCCAA and TTTGCCCTAAGCTCATCTCGT for Nrf2; CCATGTTCACCAACGGGCTTC and CGTGCAGGACACACTTCTCG for Keap1; TTCCCAAATCAGCCCCGAT and TGCCATGTCAACTGCACTTCT for GCLM; TGCACATCTACCACGCAGTC and ATCGCCTCCATTCAGTAACAAC for GCLC; ATGCTATGAACTTCAACCCCATC and TTCCAGCTTCTTGTGTTCGG for NQO-1; CACATCCAAGCCGAGAATGC and GCTTGTTGCGCTCTATCTCC for HMOX-1; and ATCCGTAAAGACCTCTATGCC and CTCCTGCTTGCTGATCCAC for β-actin. The PCR reaction system including 1 μL of cDNA, 0.4 μM of forward and reverse primers, and 10 μL of TB Green *Premix Ex Taq* II, was finally adjusted to the required total volume of 20 μL by adding RNase free water. The real-time PCR (Lightcycler 96, Roche, Switzerland) cycling conditions were 95 °C for 5 min, followed by 45 cycles of 95 °C for 10s, 59 °C for 10s, 72 °C for 10 s; (Roche, Switzerland). Following amplification, the melting curves were tested by slowly heating from 60 °C to 95 °C in increments of 0.5°C/s. During this process, we used continuous fluorescence collection to confirm that the peak signal was produced only from the target genes. Relative quantification methods (2^-ΔΔCt) were used to calculate the relative transcriptional level of each gene according to the Cycle threshold value (Ct), in which the expression of Nrf2 and Keap1 genes was normalized against that of *β*-actin.

### 2.6. Statistical Analysis

Statistical analysis was performed using IBM SPSS Statistics version 22 (IBM, Armonk, NY, USA). The values obtained from biological samples were analyzed using a one-way ANOVA, followed by multiple comparisons using *post hoc* Tukey test. The homogeneity among different groups was analyzed; if heterogeneity presented among the different groups, the Games–Howell model was utilized. Results are expressed as mean ± standard deviation (SD) for the eight mice in each group (*n* = 8), or exhibited as Box and Whisker plots, and values of *p* < 0.05 considered as statistically significant.

## 3. Results

### 3.1. Antioxidant Enzymes in Plasma or Muscle

**Figure 2:**
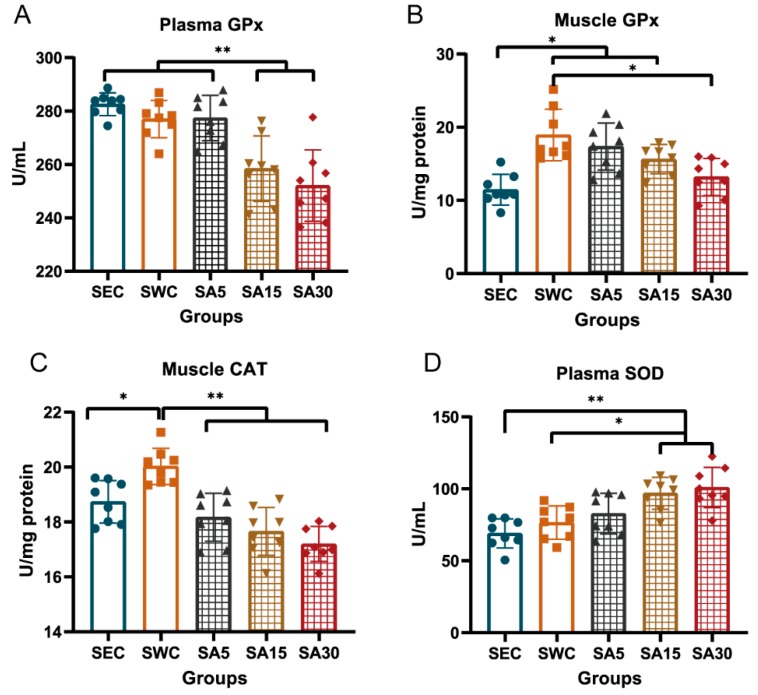
Effects of astaxanthin on the GPx, CAT, and SOD activity in swimming training mice. (**A**) and (**B**) display GPx activity in plasma and gastrocnemius muscle among different groups, respectively. (**C**) exhibits CAT activity in gastrocnemius muscle among various groups, respectively. (**D**) shows SOD activity in plasma among different groups Values are means ± SD (*n* = 8). The symbol * indicates a significant difference at *p* < 0.05, and ** indicates a significant difference at *p <* 0.01.

Supplementation of ASX decreased plasma and skeletal muscle GPx and CAT, but increased plasma SOD (Figure 2). It is suggested that supplementation of 15 mg and 30 mg/kg BW of ASX decreased the plasma GPx activity in swimming training mice, compared with that in the SWC groups (*p* < 0.001 and *p =* 0.013, respectively) and SEC group (*p <* 0.001), However, the dose of 5 mg/kg BW seemed not to affect GPx in the training mice, compared with the two higher dose groups (Figure 2A). In addition, the muscle GPx activity of the SWC group was higher than that of the SEC groups (*p =* 0.005). Meanwhile, the GPx activity in the SA5 and SA15 groups were higher than in the SEC group (*p =* 0.001, and *p <* 0.001, respectively), and the activity in the SA30 group was significantly lower than that of the SWC group (*p =* 0.002), and almost approached the level of the SEC groups (*p =* 0.711; Figure 2B). In addition, it was observed that the CAT activity in the SWC group was higher than that of the SEC group (*p* = 0.024), and supplementation of three different doses (5, 15, and 30 mg/kg BW) of ASX decreased CAT level in the training mice, compared with the SWC group (*p <* 0.001; Figure 2C). Meanwhile, the highest dose (30 mg/kg BW) of ASX significantly inhibited CAT activity, which was lower than that observed in the SEC group (*p =* 0.014). However, the SOD activity among the different groups demonstrated a gradually increasing trend (Figure 2D). It was suggested that the SOD activity increased in the SA15 and SA30 groups, compared with the SEC and SWC groups (*p* < 0.05).

### 3.2. Plasma Malondialdehyde and Creatine Kinase and Muscle Nrf2-Keap1 Transcription

**Figure 3:**
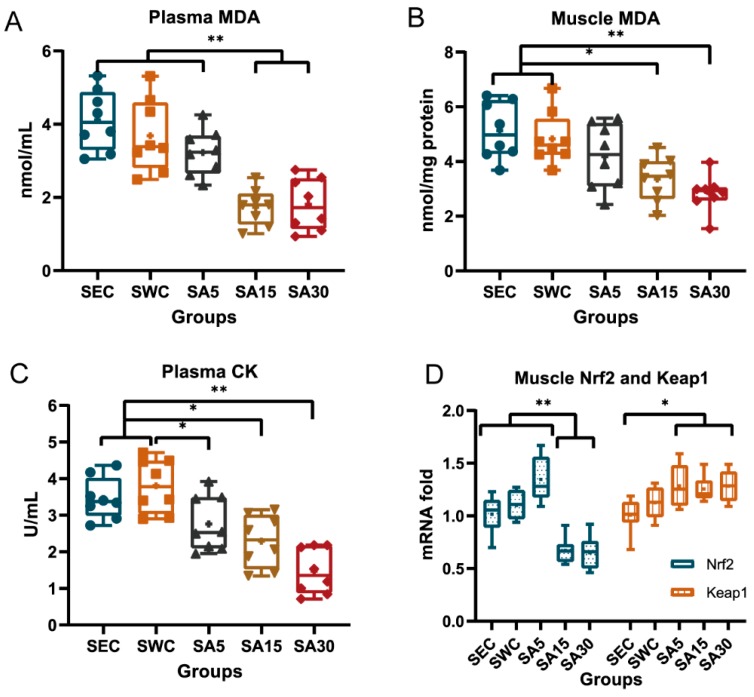
Effects of astaxanthin on the MDA level, CK activity, and Nrf2-Keap1 factors’ transcriptional level in swimming training mice. (**A**) and (**B**) show the amount of MDA in plasma and muscle among different groups, respectively. (**C**) represents CK activity in plasma among different groups. (**D**) outlines transcriptional levels of Nrf2 and Keap1 factors among different groups, in which the fold changes are expressed in relation to the mean of the SEC group. Values are expressed as Box-and-Whisker plots, in which the bottom and top of the box present the first and third quartile, respectively; the band inside the box is always the second quartile (the median); lines extending vertically from the boxes (whiskers) stand for the upper and lower extreme (the highest and lowest number in a set of data), and plus signs indicate the mean for each group. The symbol * indicates a significant difference at *p* < 0.05, and ** indicates a significant difference at *p* < 0.01.

Supplementation of ASX decreased MDA and CK levels in mice plasma or skeletal muscle and inhibited the transcriptional level of Nrf2 in gastrocnemius muscle (Figure 3). Both plasma and gastrocnemius MDA decreased in the SA15 and SA30 groups, compared with the SWC group (*p <* 0.001) and the SEC group (*p <* 0.001). However, the group treated with 5 mg/kg BW of ASX was not shown to decrease plasma and gastrocnemius MDA, which were higher than that in the groups administrated with 15 and 30 mg/kg BW of ASX (*p* < 0.01; Figure 3A,B). Similarly, administration of ASX decreased the CK levels in swimming training mice (Figure 3C). Following mutiple comparison, the results showed that the groups supplemented with 5, 15, and 30 mg/kg BW of ASX decreased plasma CK value, compared with the SWC group (*p* = 0.031, *p* = 0.001, and *p* < 0.001, respectively). Meanwhile, the CK value in the SA15 and the SA30 groups was lower than in the SEC group (*p* = 0.012 and *p* < 0.001, respectively). In addition, the higher dose of ASX exhibited a greater inhibitory effect on CK and the value in SA30 group was even lower than that of the SA5 group (*p* = 0.005). 

Additionally, supplementation of 15 or 30 mg/kg BW of ASX downregulated the transcriptional level of Nrf2 by 41% and 39% in the gastrocnemius muscle, compared with the SWC group (*p <* 0.001; Figure 3D). Meanwhile, the Keap1 level of three SA groups remained unchanged, compared with the SWC groups (*p* > 0.05) but was higher than that of the SEC group (*p =* 0.029, *p =* 0.034 and *p =* 0.015, respectively). 

### 3.3. mRNA of Nrf2 and Nrf2-Dependent Enzymes in Heart

**Figure 4:**
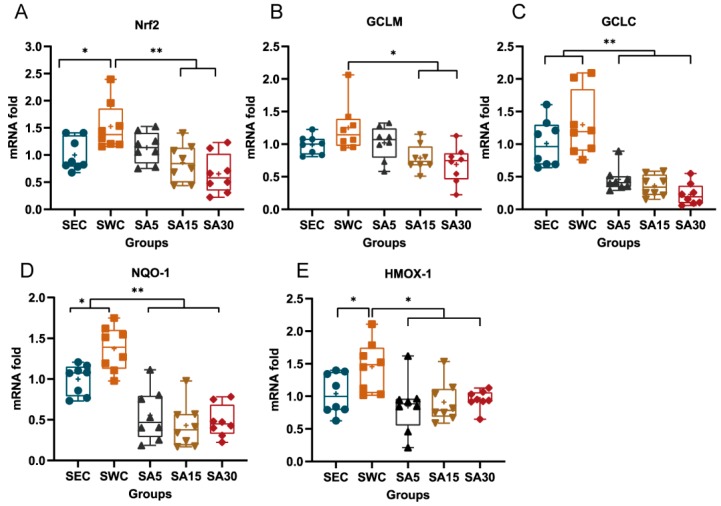
Effects of astaxanthin on transcription of Nrf2 and Nrf2-dependent enzymes in swimming training mice heart. (**A**) exhibits mRNA levels of Nrf2 among different groups, (**B**) presents GCLM transcriptional levels among different groups and (**C**) exhibits GCLC transcriptional levels among different groups. (**D**) shows NQO-1 transcriptional level among different groups and (**E**) shows HMOX-1 transcriptional level among different groups. Values are expressed as Box-and-Whisker plots, in which the bottom and top of the box present the first and third quartile, respectively; the band inside the box is always the second quartile (the median); lines extending vertically from the boxes (whiskers) stand for the upper and lower extreme (the highest and lowest number in a set of data), and plus signs indicate the mean for each group. The symbol * indicates a significant difference at *p* < 0.05, and ** indicates a significant difference at *p* < 0.01.

The mRNA level of Nrf2 in the SWC group elevated by 1.5-fold after exercise, compared with the sedentary group (*p* = 0.036). The mRNA levels of Nrf2 in the SA15 and SA30 groups were shown to be decreased, compared with the SWC group (*p* < 0.01; Figure 4A). Accordingly, the transcriptional level of GCLM and GCLC was suggested to be decreased in astaxanthin supplement groups. It was observed that the mRNA levels of GCLM among SA15 and SA30 groups significantly decreased, compared with the SWC group (*p* < 0.01; Figure 4B). Meanwhile, the mRNA levels of GCLC among the SA5, SA15, and SA30 groups were significantly lower than the SWC and SEC groups (*p* < 0.05; Figure 4C). Additionally, NQO-1 was inhibited as well after supplementation of astaxanthin. The mRNA level of NQO-among the SA5, SA5, and SA30 groups significantly decreased, compared with the SWC group (*p* < 0.01), meanwhile, the mRNA level in the SWC group was higher than the SEC group (*p* > 0.05; Figure 4D). Meanwhile, the transcriptional level of HMOX-1 was significantly increased in the SWC group, compared with the SEC group. Meanwhile, the level in the SA groups decreased in comparison to the SWC group (Figure 4E)

### 3.4. Plasma Nitric Oxide Synthase 

**Figure 5:**
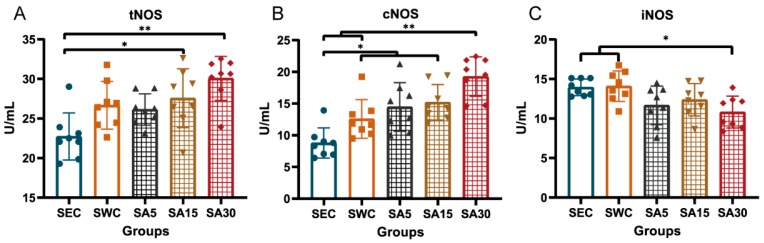
Effects of astaxanthin on NOS activity in plasma in swimming training mice. (**A**) exhibits tNOS levels among different groups, (**B**) exhibits cNOS levels among different groups, and (**C**) presents iNOS level among different groups. Values are means ± SD (*n* = 8). The symbol * indicates a significant difference at *p* < 0.05, and ** indicates a significant difference at *p* < 0.01.

We found that supplementation of ASX affected NOS level in plasma. It was demonstrated that supplementation of 15 and 30 mg/kg BW of ASX increased the tNOS level in swimming training mice, compared with the SEC group (*p* = 0.03 and *p* < 0.001, respectively; Figure 5A). It was also noted that as the ASX dose proportionally increased, the cNOS activity among the different groups demonstrated an increasing trend (Figure 5B). We found that only the group supplemented with 30 mg/kg BW of ASX increased cNOS level, compared with the SWC group (*p* = 0.007). Meanwhile, the cNOS activity in all the three SA groups was higher than in the SEC group (*p* = 0.012, *p =* 0.004, and *p* < 0.001, respectively). In addition, as the ASX dose increased, the iNOS activity proportionately decreased (Figure 5C). The results showed that the iNOS decreased in the SA30 group, compared with the SEC and SWC groups (*p* = 0.035 and *p* = 0.044, respectively).

## 4. Discussion

The primary finding from our work demonstrates that following administration of ASX and moderate-intensity swimming training, high-dose ASX has the potential to suppress oxidative stress and associated enzymatic antioxidants. There is now substantial evidence postulating that RONS are essential signaling molecules driving cellular adaptations [34,35,36,37,38]. Indeed, recent work suggests that exercise-induced oxidative stress may be a critical component for adaption to endurance training [39]. Our findings demonstrate that swimming training in mice treated with 15 mg or 30 mg/kg BW of ASX decreases antioxidant activity and oxidative stress. Previous studies report that antioxidant supplementation blunts the expression of key antioxidant enzymes and hampers exercise adaption. According to Gomes-Cabrera et al., supplementation of 500 mg/kg BW of Vitamin C combined with exercise training decreases the expression of Mn-SOD and GPx in mouse skeletal muscle and significantly inhibited mitochondrial factors, including peroxisome proliferator-activated receptor gamma coactivator 1-alpha (PGC-1α), Nuclear respiratory factor 1 (NRF-1), and mitochondrial transcription factor A (MTF-A), and hampered endurance adaptions [16]. Similarly, Meier et al. found that supplementation of an antioxidant cocktail (coenzyme Q10, 1% N-acetyl-cysteine, and vitamin C) inhibited the expression of SOD, GPx, and CAT in skeletal muscle of exercise-trained mice [17]. Our findings suggest that supplementation of 15 or 30 mg/kg BW of ASX in mice performing the moderate-intensity exercise significantly decreases in GPx and CAT activity in plasma or muscle. Furthermore, we found that 15 and 30 mg/kg BW of ASX also decreased Nrf2 transcription in skeletal muscle, but keap1 remained unchanged. This suggests that the expression of antioxidant enzymes is perhaps suppressed partially via downregulation of the transcription of Nrf2. Because Nrf2 upregulates the expression of antioxidant enzyme genes by acting on antioxidant response element (ARE) and keap1 anchors Nrf2, which will be ubiquitinated and then decomposed [40,41]. Meanwhile, we also observed that heart Nrf2 is downregulated in ASX-administrated groups. Moreover, Nrf2-dependent genes GCLC and GCLM, which are the first-rate limiting enzymes of glutathione synthesis, were also downregulated. Two studies showed that ASX supplementation increased the proportion of glutathione (GSH) content in mice soleus muscle, which was considered as basal storage of antioxidative capacity [29,42]. Although we did not quantify GSH in our study, we observed that mRNA of GCLC and GCLM in heart decreased in the ASX-treated groups. The mRNA levels of both NQO-1 and HMOX-1 also decreased in heart among the ASX-administrated groups in comparison to the SWC group, suggesting the amount of quinones and hemes generated in heart are not necessary to trigger the higher-level expression of NQO-1 and HMOX-1.

In addition, plasma SOD activity increased in the medium and high ASX dosage groups. The plasma SOD is mainly composed of SOD3 (EC-SOD), which exerts an important protective role in the vascular wall, and it was documented that the vasoactive factors such as histamine, vasopressin, oxitocyn, endothelin-1, serotonin, and heparin markedly increased the enzyme level in cultured arterial smooth muscle [43]. Previous report showed that SOD3 in alveolar type II pneumocytes was upregulated by TNF-α and INF-Υ through activation of nuclear factor Kappa-B (NF-κB). Further, exercise training increases nitric oxide in mouse vessel endothelial cells, which in turn upregulates expression of SOD3 in adjacent smooth muscle cells [44]. Meanwhile, ASX did not directly scavenge superoxide anion free radicals [24]. The elevated concentration of SOD3 prevents the degradation of NO by oxygen radicals. The nitric oxide system is a fundamental determinant of cardiovascular homeostasis and regulates systemic blood pressure, vascular remodeling, and angiogenesis [45]. We found the plasma constitutive NOS (cNOS) activity was increased in the SA30 group. The cNOS consists of two isoforms: epithelial NOS (eNOS) and neuronal NOS (nNOS) [46], but in the plasma, it is mainly composed of eNOS that are produced by epithelial cells [47]. Moncada et al., reported that nitric oxide (NO) is an endothelium-derived relaxing factor, which contributed to the beneficial effects of exercise on the cardiovascular system [48]. Furthermore, Sessa et al., has shown that chronic exercise in dogs increased coronary vascular nitric oxide production and eNOS expression [49]. Other studies in rats and humans have shown that nitric oxide contributes to glucose uptake and increased skeletal muscle basal glucose disposal [50]. From the above studies, and in line with our data, we concur that ASX can potentially stimulate eNOS activity, and this may facilitate vascular relaxation or indeed contribute to improved recovery after chronic exercise. The increase in plasma iNOS can also be considered as a marker of stimulated inflammatory status [51]. We observed a decrease in iNOS following ASX supplementation in the swimming trained mice. Lee et al., revealed that supplementation of ASX can prevent iNOS expression by blocking expression of proinflammatory genes along with NF-κB [52].

Malondialdehyde (MDA) mainly results from lipid peroxidation of polyunsaturated fatty acids [53]. We observed that administration of 15 and 30 mg/kg BW of ASX significantly decreased plasma or muscle MDA level but there were no significant effects on MDA level following the 5 mg/kg BW dose. Other studies have shown that ASX supplementation can decrease MDA level in addition to blunting antioxidant enzyme activity [29,42,54]. However, there were no substantial changes in MDA in both the placebo and ASX groups in elite young soccer players after 90 days of supplementation [55]. 

The efflux of muscle CK is an indication of a change to normal membrane structure possibly induced by muscle damage; this increased membrane permeability results in CK leakage into the extracellular space [56]. A previous study suggested that chronic exercise increased CK level in mice plasma [57]. In our study, we found that the CK activity agrees with the profile recorded for MDA. Supplementation of three different dosages of ASX significantly decreased plasma CK, compared with the swimming control group. Other studies provide evidence that ASX can decrease CK activity in mice [26,57] or in humans [55,58]. Nevertheless, Richard et al., reported that supplementation of 4 mg/day ASX in humans did not favorably affect CK values associated with skeletal muscle injury following eccentric resistance strength training [59]. Klinkenberg et al., demonstrated that supplementation of 20 mg/day ASX did not significantly decrease plasma CK in well-trained cyclists [60]. ASX is well defined as an efficient scavenger of ROO• and RO• radicals, which are reactive promoters of lipid peroxidation [24]. Thus, ASX has the potential to inhibit lipid peroxidation on the cell membrane and decrease oxidative stress following chronic exercise (as partially observed by the reduction in MDA in our study).

In summary, 5 mg/kg BW of ASX supplementation did not reduce GPx and CAT activity, nor did it suppress expression of Nrf2 in exercise trained mice. However, as the ASX supplemental dosages were raised to 15 mg/kg BW and 30 mg/kg BW, it seemed to decrease the GPx and CAT level as well as Nrf2 in the moderate-intensity swimming training mice. Therefore, using higher dosages of ASX in moderate-intensity exercise may blunt the expression of antioxidant enzymes. However, in high-intensity exercise, cells can generate an overabundance of RONS, and this may require a higher production of antioxidants to neutralize and balance oxidative stress. This may be a potential reason why there is a dosage-dependent relationship between duration time and ASX supplementation in the exhaustive training mice [28]. In our view, the proper dosage used in exercise may optimize the training effects by protecting against exercise-induced RONS overproduction; however, overdosed supplementation may impair the beneficial effects from exercise. Currently, the absorptivity of astaxanthin between humans and mice is not widely studied, thus we cannot estimate the relative amount for humans. The safe dosage used for human consumption has been discussed in a previous review, and no adverse effects have been observed when humans were supplemented with dosages ranging from 2.38 to 40 mg/day [61]. Nevertheless, the optimal dosage of ASX supplementation in accordance with different oxidative stress for human health benefits needs further investigation in the future. Finally, there are limitations of this study. Firstly, the physiological status of the exercising animals was not measured due to a lack of appropriate equipment. Secondly, the effects of ASX on the mice only receiving ASX treatment were not evaluated. Moreover, due to financial restrictions, we did not collect a complete set of redox biology indices in tissue or plasma. Lastly, since antioxidant enzymes are predominantly present and most active in the intracellular environment (i.e., CuZnSOD, MnSOD), there may be a differential concentration between sites of determination (i.e., intracellular vs. extracellular), and it is conceivable that the analytical kits have limitations in accurate plasma and muscle quantification. Therefore, in any future investigation, protein levels of selected antioxidant enzymes should be included to further evaluate the effects of ASX on exercise.

## 5. Conclusions

The findings from this study indicate for the first time that high dosage of astaxanthin suppresses GPx and CAT activity in plasma or muscle in moderate-intensity training mice, and downregulates the transcription of Nrf2 and Nrf2-dependent enzymes in skeletal muslce or heart, along with attenuating plasma and muscle MDA.

## Supplementary Materials

The following are available online at https://www.mdpi.com/2072-6643/11/6/1244/s1, S1: The mouse feed composition used in this study.
