# Supplementary material for: High-Dose Astaxanthin Supplementation Suppresses Antioxidant Enzyme Activity during Moderate-Intensity Swimming Training in Mice
Source: Nutrients. 2019 May 31;11(6):1244. doi: 10.3390/nu11061244 (PMC6627865; doi:10.3390/nu11061244)
Supplement: Supplementary file 1 [file nutrients-11-01244-s001.pdf]

The composition of Mouse feed

Macronutrients

Ingredient	Content
Crude protein	≥ 14%
Crude fat	≥ 8%
Crude fibre	≤ 9%
Total	3400
Clarories	kcal/kg

Amino acids

Ingredient	Content
Arg;	≥ 0.90 %
Lys	≥ 0.85 %
Met	≥ 0.35 %
Cys	≥ 0.25 %
Trp	≥ 0.20 %
Gly	≥ 0.95 %
His	≥ 0.38 %
Leu	≥1.40 %
Ile	≥ 0.95 %
Phe	≥ 0.85 %
Tyr	≥ 0.60 %
Thr	≥ 0.65 %
Val	≥ 0.90 %

Vitamins

Ingredient	Content
Vitamin A	≥ 1 IU/g
Vitamin D	≥ 6.8 IU/g
Vitamin E	≥ 83 ppm
Vitamin K	≥ 1.0 ppm
Thiamine	≥ 15 ppm
Riboflavin	≥ 6.9 ppm
Niacin	≥ 75 ppm
Pantothenic acid	≥ 27 ppm
Choline	≥ 2950 ppm
Pyridoxine	≥ 12 ppm
Folic acid	≥ 1.9 ppm
Biotin	≥ 0.28 ppm
Cobalamin	≥ 0.048 ppm

Minerals

Ingredient	Content
Ca	≥ 9.4%
P	≥ 5.8 %
K	≥ 0.68 %
Na	≥ 0.18 %
Mg	≥ 0.18 %
Fe	≥ 160 ppm
Zn	≥ 48 ppm
Mn	≥ 53 ppm
Cu	≥ 5.8 ppm
I	≥ 0.6 ppm
Cr	≥ 1.2 ppm
